# Targeting TLR4 Signaling to Blunt Viral-Mediated Acute Lung Injury

**DOI:** 10.3389/fimmu.2021.705080

**Published:** 2021-07-02

**Authors:** Kari Ann Shirey, Jorge C. G. Blanco, Stefanie N. Vogel

**Affiliations:** ^1^ Department of Microbiology and Immunology, School of Medicine, University of Maryland, Baltimore, MD, United States; ^2^ Sigmovir Biosystems, Inc., Rockville, MD, United States

**Keywords:** TLR4, influenza, ALI, viruses, HMGB1

## Abstract

Respiratory viral infections have been a long-standing global burden ranging from seasonal recurrences to the unexpected pandemics. The yearly hospitalizations from seasonal viruses such as influenza can fluctuate greatly depending on the circulating strain(s) and the congruency with the predicted strains used for the yearly vaccine formulation, which often are not predicted accurately. While antiviral agents are available against influenza, efficacy is limited due to a temporal disconnect between the time of infection and symptom development and viral resistance. Uncontrolled, influenza infections can lead to a severe inflammatory response initiated by pathogen-associated molecular patterns (PAMPs) or host-derived danger-associated molecular patterns (DAMPs) that ultimately signal through pattern recognition receptors (PRRs). Overall, these pathogen-host interactions result in a local cytokine storm leading to acute lung injury (ALI) or the more severe acute respiratory distress syndrome (ARDS) with concomitant systemic involvement and more severe, life threatening consequences. In addition to traditional antiviral treatments, blocking the host’s innate immune response may provide a more viable approach to combat these infectious pathogens. The SARS-CoV-2 pandemic illustrates a critical need for novel treatments to counteract the ALI and ARDS that has caused the deaths of millions worldwide. This review will examine how antagonizing TLR4 signaling has been effective experimentally in ameliorating ALI and lethal infection in challenge models triggered not only by influenza, but also by other ALI-inducing viruses.

## Summary sentence

This review focuses on the role of TLR4 in the development of virus-induced acute lung injury (ALI) and its potential for therapeutic targeting.

## Introduction

Many pathogens mutate rapidly, leading to anti-microbial resistance or altered expression of immunogenic epitopes such that extant vaccines or therapeutic drugs are rendered ineffective. The ability of the host to recognize and respond to immunologic “danger” is the result of the exposure of the host to a pathogen’s unique components known as “pathogen-associated molecular patterns” (PAMP) that are readily detected by host surveillance receptors. Such “pattern recognition receptors” (PRR) (1) are expressed on cells of the innate immune system and their activation initiates intracellular signaling and transcriptional programs that lead to a rapid and strong primary response against the pathogen, *e.g*., Gram-negative lipopolysaccharide (LPS) interacts with Toll-like receptor 4 (TLR4) leading to the induction of thousands of genes, many of which are proinflammatory (2). Also, as part of this rapid response, host-derived molecules are often released during cellular necrosis or are secreted upon cellular stress. These molecules are collectively referred to as “danger-associated molecular patterns” (DAMPs) (1), and activate identical PRR signaling pathways, *e.g*., HMGB1 is a chromatin-associated protein that, when actively secreted or released from dying cells (3), engages TLR4 through its co-receptor, MD-2, and elicits a pro-inflammatory response similar to that of LPS (4). 

## TLR Signaling

In humans, Toll-like receptors (TLRs) are a closely related family of 10 transmembrane PRRs that sense unique microbial chemistries or PAMPs and/or host-derived DAMPs (5, 6). Ligand interaction(s) with a TLR’s N-terminal leucine-rich repeat (LRR) domain results in TLR dimerization, leading to conformational changes that cause interaction of C-terminal TLR “Toll-IL-1 receptor resistance (TIR)” domains to form a molecular scaffold (7) that facilitates recruitment of adaptor molecules (*e.g.*, TIRAP/MyD88 or TRIF/TRAM) through TIR-TIR interactions. In turn, docking of MyD88 or TRIF to the TLR TIR domain permits further recruitment of downstream signaling proteins to form a large molecular complex called a Myddosome or Triffosome, respectively (8). The MyD88-dependent pathway is recruited by all cell surface- and endosomally-expressed TLRs except endosommaly-expressed TLR3. TLR4 recruitment of MyD88 primarily activates NF-κB, a transcription factor (TF) required for inductin of most pro-inflammatory cytokine and chemokine genes. The TRIF (MyD88-independent) pathway, that is activated only from two endosomally located TLRs (*i.e.*, TLR4 and TLR3), activates IRF3, a TF required for induction of IFN-β and other TRIF-dependent genes. However, the TRIF-mediated pathway bifurcates and also results in delayed NF-κB activation. TLR4 is unique in that (i) it requires a non-covalently associated co-receptor, MD-2, for ligand binding, and (ii) it is the only TLR that activates both MyD88 and TRIF signaling pathways (6). That Gram-negative lipopolysaccharide (LPS) activates both MyD88 and TRIF pathways through TLR4/MD-2 is thought to explain why TLR4 signaling is associated with highly inflammatory responses as seen in Gram-negative sepsis and other diseases that are mediated by “cytokine storms”.

## Influenza

New antigenic variants of influenza emerge annually, giving rise to seasonal outbreaks with significant morbidity and mortality, resulting in up to 500,000 deaths worldwide (9, 10). Hospitalization and deaths occur mainly in high-risk groups (*e.g.*, children, elderly, chronically ill). In addition, strains to which humans have no prior immunity may appear suddenly and the resulting pandemics can be catastrophic, as illustrated by the 1918 “Spanish flu” (11, 12). In March 2009, a novel influenza A virus, pandemic H1N1, emerged that contained a previously unseen combination of genes of swine origin, and caused hospitalizations and deaths in both high-risk individuals and in healthy adults and children (12, 13). While vaccination provides significant protection, the ability to predict the influenza strains to be incorporated into the following year’s vaccine sometimes fails (14, 15). Although anti-viral therapeutics are available, patients must be treated early in infection for them to be effective. Moreover, virus resistance to neuraminidase (NA) inhibitors (*i.e.*, oseltamivir, zanamivir) and M2 channel inhibitors (*i.e.*, amantadine, rimantadine) is common (16, 17). Thus, a new approach to block influenza-induced, host-mediated disease therapeutically would represent a significant therapeutic treatment against infectious diseases.

## A Central Role for TLR4 Signaling in Acute Lung Injury (ALI) caused by Influenza Infection

While influenza virus is recognized by multiple PRRs including TLR3, TLR7, TLR8, TLR10, retinoic acid-inducible gene I (RIG-I), and NOD-like receptor family pyrin domain containing 3 (NLRP3) (18), a central TLR4 signaling axis for the induction of ALI by multiple insults was proposed by Imai et al. (19). They proposed that diverse chemical or microbial insults to the lung trigger NADPH-dependent reactive oxygen species (ROS) that generate the formation of oxidized phospholipids such as oxidized 1-palmitoyl-2-arachidonoyl-phosphaticycholine (OxPAPC). In this study, wild-type mice were compared to mice with targeted mutations in genes involved in TLR signaling by inducing ALI by acid aspiration, instillation of UV-inactivated H5N1 influenza, infection with live bacterial pathogens, and aspiration of purified OxPAPC *in vivo* and confirmed *in vitro*. The authors concluded that a common TLR4-, TRIF-, and IL-6-dependent pathway to ALI was mediated by OxPAPC-activated lung macrophages. In support of the idea that this pathway is common to other infectious mediators of ALI, Imai et al. found that fixed lung sections of SARS-CoV-1 patients and from animals infected with anthrax, monkey pox, or *Yersinia pestis* also showed OxPAPC deposition (19). Shortly thereafter, Nhu et al. reported that TLR4^-/-^ mice were highly refractory to infection with mouse-adapted influenza, A/PR/8/34 (PR8) (20). Thus, it was hypothesized that blocking TLR4 signaling might represent a viable therapeutic approach for mitigating influenza-induced ALI. Therapeutic administration of an LPS analog antagonist, Eritoran (E5564) (21, 22) that acts by competing for the LPS-binding site on MD-2 (23), resulted in a dose- and time-dependent protection of mice from lethal influenza infection when administered starting on day 2 post-infection once daily for 5 consecutive days (24). Even when Eritoran was administered to PR8-infected mice starting as late as day 6 post-infection, it still resulted in statistically significant survival, as well as improved clinical score, and inhibition of histopathology, cytokine gene and cytokine protein expression. While Eritoran was shown to decrease viral titers in the lung, it was not found to be directly antiviral (24). These findings were confirmed in the cotton rat (*Sigmodon hispidus*) (24, 25), a rodent species that is uniquely susceptible to non-adapted human respiratory viruses including influenza (24, 26).

Protection of mice and cotton rats from influenza-induced ALI has since been replicated with many other structurally and mechanistically distinct TLR4 antagonists including anti-TLR4 antibodies (25, 27), a cell-permeable TLR TIR decoy peptide (2R9) (28, 29), a virus-derived peptide 9R-VIPER (25, 30), a small molecule TLR4 antagonist, TAK242 (31, 32), another LPS analog antagonist (FP7) (33, 34), and the theta defensin, RC-101 (35). The “lung leak” caused by PR8 infection of mice was shown to be reversed by Eritoran administration, as well as by treatment with AT-1001 (larazotide acetate), a peptide that blocks LPS-induced tight junction disassembly in the lung (36). The blocking of TLR4 to protect against more lethal strains of influenza has also been reported. Xu et al. showed that both Eritoran (administered 2 h post-infection by intravenous inoculation), as well as another TLR4 inhibitor, epigallocatechin-3-gallate [EGCG (37)], a polyphenol naturally found in green tea (38), prolonged survival of mice infected with H9N2 influenza when administered after infection by oral gavage, and that treatment with Eritoran or EGCG diminished H9N2-induced ALI (39). While this study also showed that EGCG reduced viral titers (similar to that observed by treatment with Eritoran (24, 39), it did not assess whether EGCG was directly antiviral; however, another study reported that EGCG had greater antiviral activity against influenza B virus than influenza A virus (40). Together, these data strongly support the concept that influenza infection triggers TLR4-mediated inflammation leading to ALI.

While the TLR4/TRIF signaling axis was suggested to be central for ALI, MyD88 has also been implicated in the host response to influenza. In survival studies, MyD88^-/-^ mice were shown to have higher mortality rates compared to wild-type and TRIF^-/-^ mice (41). Additionally, MyD88 deficiency resulted in significantly higher lung pathology and diminished the cytokine response to influenza after infection when compared to wildtype mice or TRIF^-/-^ mice (41, 42). The role of IRAK4, the first enzyme recruited to MyD88 for signaling, was also examined during influenza infection. IRAK4 kinase-dead knock-in (IRAK4^KDKI^) mice, which have a catalytically inactive form of IRAK4 that precludes MyD88-dependent signaling, were shown to be susceptible to influenza with a slight delay in the meantime to death compared to wildtype mice. However, TRIF^-/-^ mice infected with influenza showed less mortality compared to wildtype mice (25). Treatment with TLR4 antagonists such as Eritoran, FP7, and TAK-242 blocked both MyD88- and TRIF-dependent pathways (24, 32, 34). While MyD88^-/-^ mice were found to be more susceptible to primary influenza infection than wildtype control mice, they were protected from a secondary influenza challenge after a low dose primary infection (41) suggesting that MyD88 was not required for protection against a secondary influenza infection. Eritoran treatment after lethal influenza challenge of wildtype mice, which inhibits both MyD88- and TRIF-dependent signaling, did not block the ability to mount an adaptive immune response to a secondary lethal influenza challenge (25). Taken together, these studies show both MyD88- and TRIF-dependent pathways contribute to influenza-induced disease.

As mentioned, influenza can induce oxidative stress by production of oxidized phospholipids (19) and reactive oxygen species (43). N-acetyl-L-cystine (NAC), a potent antioxidant (44), was also shown to protect mice against lethal H9N2 swine influenza infection. NAC, when administered prophylactically 1 h prior to infection and every 4.5 h thereafter for 5 treatments, interfered with viral replication, thereby improving survival. Along with oseltamivir treatment, NAC also reduced disease severity (45). This study also showed that rather than inhibiting TLR4 signaling, NAC inhibited expression of TLR4 (45). While this may be true, an earlier study showed that NAC also was able to inhibit influenza viral replication *in vitro* in A549 cells (46), suggesting multiple mechanisms by which NAC may contribute to host protection against influenza infection.

Kaempferol, a common flavonoid with known anti-inflammatory properties and anti-oxidative effects (47–50), has been shown to decrease LPS-induced TNF-α and IL-1β in macrophages (51–53). Treatment of H9N2-infected BALB/c mice with Kaempferol every 12 h starting 1 h prior to infection, resulted in improved survival, with reduced lung pathology, decreased pulmonary edema, cytokine production, and decreased viral titer in the lungs (54). While this study did not directly analyze whether Kaempferol is directly antiviral, another study showed that Kaempferol was more potent as an antiviral against influenza B virus than influenza A virus and that the mechanism was likely to work by binding to glycoproteins of the viral envelope to inhibit viral replication (40). Like NAC, Kaempferol may work both as an antiviral as well as *via* inhibition of TLR4 expression and thereby inhibit the TLR4 signaling pathway and inflammatory responses.

Curcumin, a polyphenol component of turmeric (55), has been widely studied for its anti-inflammatory, anti-oxidative, and other pharmacological effects (56). Due to problems with solubility and bioavailability, curcumin analogs have been developed (56, 57). Both curcumin and its analogs have been shown to block TLR4 signaling during LPS-induced sepsis and ALI through various mechanisms including binding MD-2 and inhibiting NF-κB or ERK activation (58–60). Curcumin, and a structural analog, monoacetylcurcumin (MAC), have been assessed for their effectiveness during influenza infection (61–64). Curcumin was shown to inhibit replication of multiple strains of IAV *in vitro* by disrupting viral attachment to cells by inhibiting hemagglutinin activity of the virus (61, 62, 64). When mice were administered curcumin twice daily for 6 days starting 24 h after influenza infection, the survival rate was significantly increased, with 150 µg/mouse of curcumin eliciting similar survival as the oseltamivir-treated control group (64). Curcumin was found to be more broad-spectrum in its effects on PRR signaling. A549 cells infected with influenza and treated with curcumin showed inhibition of influenza-induced expression of TLR2, TLR4, and TLR7, as well as inhibition of activation of MyD88- and TRIF-dependent signaling pathways (64). Taken together, curcumin can act as both a direct antiviral as well as protecting the host by inhibiting not just TLR4 signaling, but other TLRs as well.

Liu Shen Wan (LSW), a traditional Chinese medicine comprised of multiple mineral- and animal-derived components (65), has wide pharmacological effects and has been used to treat a variety of ailments for many years (66–68). LSW was shown to improve survival in a rodent model of sepsis induced by cecal ligation puncture by reducing TNF-α, but not IL-1 levels, as well as plasma malondialdehyde (MDA) contents (65). Ma et al. recently reported that LSW treatment had an anti-viral effect on influenza infection both *in vitro* as well as *in vivo* (69). LSW was found to decrease proinflammatory cytokine production in infected cells through the inhibition of the TLR4/NF-κB axis (69). When administered orally after influenza A/PR/8/34 influenza infection, LSW showed a dose-dependent increase in survival, but not as significant as treatment with oseltamivir (69). The specifics of the timing of LSW administration was not reported in this study apart from the fact that it was administered for 5 days, so the whether the LSW acts as a direct antiviral agent and/or interferes with PRR-mediated signaling remains to be determined.

Sulforaphane (SFN) is a naturally occurring compound in cruciferous vegetables such as broccoli, brussels sprouts, and cabbages (70, 71). SFN has become of interest for its use as a health supplement due to its pleiotropic effects on the immune system (71). SFN acts in an anti-inflammatory manner by suppressing TLR4 oligomerization (72), as well as by binding to MD-2 and blocking the interaction of LPS with the TLR4/MD-2 complex (70). In addition to blocking TLR4, SFN’s anti-inflammatory capabilities have been attributed to its ability to activate the anti-inflammatory TF, Nrf2, to inhibit NF-κB-induced pro-inflammatory cytokines (71). However, there are reports of cross-talk between TLRs and Nrf2 (73). Huang et al. reported that Nrf2 activation resulted in decreased TLR4-induced inflammation in the liver in an ischemia/reperfusion model (74). Several studies have examined the effects of SFN on influenza infection. In combination with β-glucan, also shown to attenuate TLR4-mediated cytokine production (75), SFN administered as an oral prophylactic supplement two weeks prior to influenza H5N1 challenge of mice, resulted in significant survival (76). However, this treatment did not decrease lung cytokine levels, but rather, increased in IFN-*γ*, IL-1β, and TNF-α compared to the control group (76). Importantly, the prophylactic treatment of mice with glucan-SFN also reduced viral titers in the lung (76), but this is possibly due to SFN acting on Nrf2 and blocking viral entry into and replication in epithelial cells (77, 78). To our knowledge, SFN has not been tested therapeutically.

**Table I** summarizes the various TLR4 antagonists and signaling inhibitors that have been shown to be efficacious against influenza infection in animal models and their mechanisms of action. The diversity of these various inhibitors of TLR4 signaling, coupled with the spectrum of mechanisms by which they act, strongly support the notion that TLR4 is key to the host inflammatory response to influenza infection.

**Table I:** Summary of agents used to treat influenza infection in rodent models.

Agent	Structure	Mechanism of Action	Timing of treatment	Direct antiviral activity	Comments
Eritoran (E5564)	Lipid A analog antagonist (21, 22)	Binds in deep hydrophobic pocket of MD-2 and competitively inhibits LPS and other TLR4 agonists (23)	Therapeutically starting days 2, 4, and 6 post-infection for 5 consecutive days.	No	Therapeutic efficacy initially demonstrated for mouse-adapted influenza strains and later in cotton rats challenged with non-adapted human influenza strains; blocks release of HMGB1 and cytokines (24, 25). Later, Eritoran demonstrated therapeutic efficacy against infection with EBOV (79)
Anti-TLR4 antibodies	Rabbit anti-mouse TLR4 antibody (27)	Binds to TLR4 and prevents activation by TLR4 ligands (27)	Therapeutically on days 2 and 4 post-infection with 1 or 2 administrations	No	Therapeutic efficacy when administered either once or twice after PR8 infection (25)
FP7	Lipid A analog antagonist (34)	Binds in deep hydrophobic pocket of MD-2 and competitively inhibits LPS (33)	Therapeutically starting on day 2 post-infection for 5 consecutive days	No	Similar to Eritoran (34)
2R9	Cell-permeable decoy peptide based on TLR2 TIR sequence (29)	Binds to TIRAP/Mal and prevents its association with TLR4 TIR (28)	Therapeutically starting on day 2 post-infection for 5 consecutive days	No	Therapeutic efficacy against mouse-adapted influenza challenge (29)
9R-VIPER	Adenovirus peptide (30)	Disrupts TLR4:TIRAP and TLR4:TRAM interactions (30)	Therapeutically starting on day 2 post-infection for 5 consecutive days	No	Partial protection against mouse-adapted influenza model (25)
TAK-242	Small molecule inhibitor (31)	Binds to TLR4 TIR domain to block MyD88-dependent signaling (31)	Therapeutically starting on day 2 post-infection for 5 consecutive days	No	Therapeutic efficacy against mouse-adapted influenza (32)
RC-101	Recombinant humanized theta defensin (80)	Inhibits TLR4 and TLR2 signaling; unknown mechanism (35)	Therapeutically starting on day 2 post-infection for 5 consecutive days	Yes^1^	Therapeutic efficacy against mouse-adapted influenza when administered for five consecutive days starting two days post-infection (35). Direct antiviral activity is not expected against influenza due to timing of therapeutic administration.
NSC77427	Small molecule peptide inhibitor (81)	Blocks action of Gastrin-Releasing Peptide (81)_;_ possibly acts by blocking TLR4-GRPR synergy (82)	Therapeutically starting on day 2 post-infection for 5 consecutive days	No	Partial protection against mouse-adapted influenza model (82)
MoAb 2A11	Highly specific anti-GRP monoclonal antibody (83)	Neutralizing antibody that binds the N-terminus of GRP (83)	Therapeutically on days 2 and 4 post-infection	No	Partial protection against mouse-adapted influenza model (82)
BW2258U89	Small molecule GRPR antagonist (84)	Inhibits GRP from binding to GRPR (84, 85)	Therapeutically starting on day 2 post-infection for 5 consecutive days	Yes	Partial protection against mouse-adapted influenza model (82)
Epigallocatechin-3-gallate (EGCG)	Polyphenol found in green tea (38)	Inhibits TLR4 signaling (38, 39)	Therapeutically^2^	Yes	Partial therapeutic efficacy against swine H9N2-infected mice (39)
N-acetyl-L-cystine (NAC)	Antioxidant (44)	Inhibits TLR4 expression (45)	Prophylactically starting 1 h prior to infection and continued every 4.5 h for 5 total treatments	Yes	Increased survival and decreased viral replication (45)
Kaempferol	Flavonoid with anti-inflammatory and anti-oxidative properties (47–50)	Inhibits TLR4 activation (54)	Prophylactically starting 1 h prior to infection and continued every 12 h for 12 total treatments	Yes	Treatment improved survival and decreased disease severity (54)
Curcumin	Polyphenol found in turmeric (55)	Inhibits TLR4-induced NF-κB activation (60)	Therapeutically starting 6 h post-infection for 6 consecutive days	Yes	Increased survival in mouse model of influenza A virus (64)
Liu Shen Wan (LSW)	Combined mineral- and animal-derivatives from pearl, realgar, borneol, toad venom, bezoar, and musk (65)	Inhibits TLR4 and NF-κB activation (68)	Therapeutically for 5 days^2^	Yes	Partial protection against mouse-adapted influenza (69)
Sulforaphane (SFN)	Natural compound found in cruciferous vegetables (70)	Inhibits TLR4 oligomerization, binds MD-2, activates Nrf2 (70, 71)	Prophylactically starting 2 weeks prior to infection for daily administration	No	Treatment increases survival and reduces lung viral titers (76)

^1^Direct antiviral activity reported for Dengue virus (86).

^2^Specific timing of administration of treatment not provided.

## Does the Timing of Treatment Play a Role in Protection?

As discussed thus far, the various compounds tested were administered either prophylactically or therapeutically (summarized in **Table I**). This leads to two questions: First, is the timing of TLR4 inhibition critical for protection? and second, do additional mechanisms contribute to protection from influenza? To address the first question, blocking TLR4 signaling therapeutically is highly effective for protection against lethal influenza infection. Delaying the start of treatment with Eritoran as late as 6 days still provided statistically significant protection (24). However, if Eritoran was administered 3 h prior to infection, and once daily for an additional 4 days, it failed to protect from lethal influenza infection (25). Interestingly, TLR4^-/-^ mice, which were shown to be refractory to influenza infection (20, 24, 25), failed to resist infection when Eritoran was administered 3 h prior to infection and once daily for 4 additional days. This suggests a non-TLR4 target, which is necessary for resistance early in infection, may also be targeted by Eritoran. CD14 has been found to facilitate TLR4, TLR2, and TLR3 signaling (87) and is required for cytokine and chemokine induction by influenza (88). In the case of TLR3, CD14 was reported to enhance TLR3-mediated signaling (89). Eritoran has been shown to bind to CD14 (24). It is possible that pre-treatment with Eritoran could therefore interfere with influenza-induced TLR3 activation early in influenza infection. Of note, CD14^-/-^ mice could not be protected by Eritoran therapy after influenza infection (24). While multiple groups and studies have shown the inhibition of TLR4 is protective during influenza infection, supporting the study by Imai et al. (19), Shinya et al. (90) reported that the TLR4-TRIF pathway was protective, rather than being detrimental, in influenza infection. This study found that pre-treatment of wildtype C57BL/6J mice with LPS prior to influenza infection promoted protection and survival (90). The upregulation of TLR3 was implicated as part of the protection, perhaps supporting the notion that CD14 enhances TLR3 signaling during influenza infection.

TLR4 antagonism by other non-TLR4 specific, more complex molecules such as naturally occurring anti-inflammatory compounds suggest that other mechanisms contribute to protection during influenza infection. NAC, Kaempferol, and SFN were all administered prophylactically, but at various times prior to infection (45, 54, 76). As stated earlier, one might postulate that the protective effects observed may reflect differences in the relative abilities of these compounds to be antiviral *vs.* host signaling modulators. In the case of SFN, the mice were given the compound for 2 weeks, well before infection (76). SFN treatment activated host Nrf2, which blocked viral entry in epithelial cells resulting in an antiviral response (77, 78). Therapeutic treatment after influenza infection with EGCG, curcumin, and LSW (39, 64, 69) also showed protection against influenza infection, but whether these responses were the result of direct antiviral activity or altered TLR4 expression and signaling will require further investigation. Regardless of timing, the inhibition of TLR4 seems to play an important role in influenza infection.

## A Central Role for DAMP-Mediated TLR4 Signaling in Acute Lung Injury (ALI) Caused by Influenza Infection

**Figure 1:**
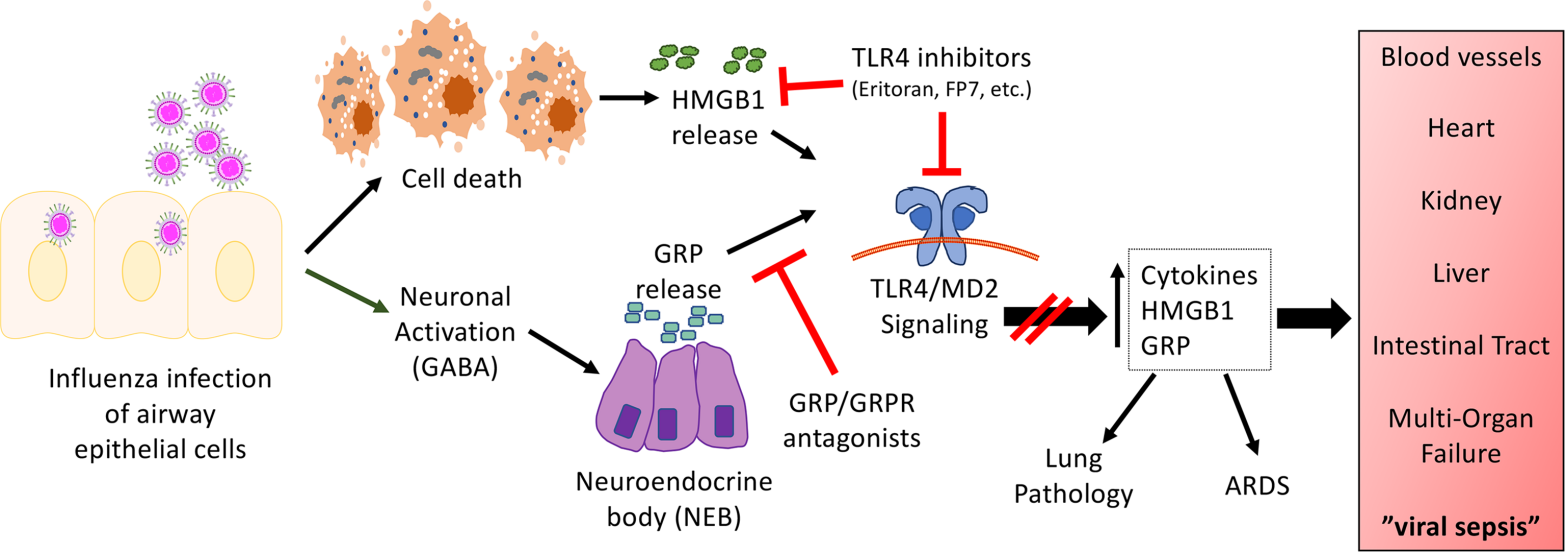
Hypothetical model for the involvement of TLR4 in influenza-induced disease. Our model proposes that influenza infects airway epithelial cells, leading to cell death and the release of DAMPs such as HMGB1. In turn, HMGB1 binds to the TLR4/MD2 complex to initiate signaling leading to release of cytokines and additional DAMPs. In addition, virus infection activates neurons in close proximity to NEBs, leading to the release of GRP. GRP has been shown to synergize with TLR4 agonists (82). Blocking either TLR4/MD2, HMGB1, or GRP mitigates influenza-induced ALI and disease pathogenesis. We hypothesize that this same mechanism is operative for other respiratory viral infections.

While all of the studies presented above support a key role for TLR4 in influenza-induced disease, influenza does not express any TLR4 PAMPs, suggesting that a host-derived DAMP produced during influenza infection might actually signal through TLR4. One such DAMP, HMGB1, engages TLR4 by binding to its co-receptor, MD-2 (4), and was shown to be produced during influenza infection in mice challenged with PR8 or in cotton rats challenged with non-adapted human influenza strains in a time-dependent manner (25, 91). Blocking HMGB1 therapeutically with a neutralizing anti-HMGB1 antibody or a small molecule inhibitor, P5779 (4), improved survival during influenza infection in mice to the same extent as Eritoran treatment (25, 92). Therapeutic administration of Eritoran during influenza infection inhibited HMGB1 release in mice and cotton rats (25). In addition to HMGB1, another host DAMP, Gastrin Releasing Peptide (GRP), was found to be produced during influenza infection with kinetics that paralleled that of HMGB1 (82). GRP has been implicated in lung inflammatory diseases including asthma, bronchopulmonary dysplasia, and hyperoxia-, ozone-, or radiation-induced lung injury (93). In the lung, GRP is produced by specialized epithelial cells, pulmonary neuroendocrine cells, that are activated by neuronally derived *γ*-aminobutyric acid (GABA) to form clusters called neuroendocrine bodies (NEB) that secrete GRP. GRP binds to its receptor, GRPR (BB2), and activates downstream signal transduction pathways (*e.g*., cAMP, MAPK, PI3K, Akt) (93, 94). GRP synergizes with LPS for cytokine production by macrophages (82). Blocking GRP therapeutically with a small molecule inhibitor, NSC77427 (81), an anti-GRP monoclonal antibody (2A11) (83), or a small molecule GRP receptor antagonist (BW2258U89) (84, 85), significantly reduced influenza-induced lethality, cytokine induction, and lung pathology in mice (82). Taken collectively, the host-derived DAMPs, HMGB1 and GRP, play important roles in mediating influenza-induced disease. The relationship between these two mediators of influenza-induced disease is currently under study. **Figure 1** represents a hypothetical model for the involvement of TLR4 and influenza-induced DAMPs that leads to influenza pathogenesis. As has been discussed inhibiting either TLR4 expression and/or DAMP-induced TLR4 signaling mitigates ALI.

Other DAMPs have been implicated in the host response to influenza. Pulmonary surfactant proteins (SP), SP-A, SP-B, SP-C, and SP-D, were initially characterized for their ability to reduce the surface tension at the air-liquid interface of the lung (95, 96). Later, these proteins were also found to be involved in the host defense of the lung, primarily SP-A and SP-D (95, 97). SP-A and SP-D have been shown to act by binding to bacteria, viruses, and fungi allowing for microbial clearance through a variety of innate immune mechanisms (97). SP-A has been shown to stimulate NF-κB activation in both human and murine macrophages *in vitro* through the TLR4 signaling pathway (98). Later, it was found that SP-A was able to directly interact with the TLR4/MD2 complex (99). SP-D was found to interact with the extracellular domains of both TLR4 and TLR2 (100), while an earlier study suggested that both SP-A and SP-D could reduce inflammatory cytokines such as TNF-α by binding CD14 and, therefore, displace LPS for signaling *in vitro* (101). Several studies have focused on the role of SP-A and SP-D during influenza infection. Both SP-A and SP-D were found to neutralize the influenza virus through different mechanisms (102, 103). While survival of mice during influenza infection was not assessed, both SP-A^-/-^ and SP-D^-/-^ mice exhibit increased susceptibility to influenza compared with wildtype mice as measured by inflammatory cytokines, pathology, and viral burden (104, 105). Interestingly, the studies of SP-A and SP-D interactions with TLRs were carried out *in vitro* only, thus their role in influenza infection *in vivo* or its interplay with TLR4 has not yet been elucidated. Nonetheless, tracheal administration of exogenous SP-D to influenza-infected SP-D^-/-^ mice at the time of infection alleviated the enhanced inflammatory response and reduced viral burden (104).

## Role of TLR4 in Secondary Bacterial Infection After Influenza

Enhanced susceptibility to secondary bacterial infection after influenza accounts for many hospitalization and deaths, particularly during pandemics (106, 107). Co-infection with bacteria usually occurs within the first or second week of influenza infection (108). Historical records indicate that during the 1918 influenza pandemic a majority of all deaths were attributed to secondary bacterial pneumonia (106), while ~34% of the 2009 pandemic infections were associated with secondary bacterial infections (107). Studies have revealed that influenza-induced IFN-β selectively blocks induction of CXCL1/CXCL2 and neutrophil activation required to counter secondary bacterial infection with *Streptococcus pneumoniae* (108). We found that antagonizing TLR4 with Eritoran therapeutically during influenza virus infection, but prior to bacterial challenge, protected mice by reversing the IFN-β-mediated suppression, resulting in enhanced chemokine production (CXCL1 and CXCL2), increased myeloperoxidase activity, and reduced bacterial burden (109). Similarly, Eritoran treatment of cotton rats infected with non-adapted human pandemic H1N1, prior to infection with multi-drug resistant *Staphylococcus aureus* (MRSA), resulted in significant amelioration of lung pathology (109). Thus, blocking TLR4 therapeutically during influenza infection not only mitigates influenza-induced disease, but also prevents an enhanced inflammatory response to secondary bacterial infection. One of the molecular mechanisms pinpointed to explain these altered transcriptional responses are suggested by the observation that IFN-β-treatment of macrophages decreased recruitment of RNA polymerase II to the promoter of the *Cxcl1* gene (109, 110). Other studies have shown similar outcomes with changes in neutrophil function after influenza that increase susceptibility to secondary bacterial infections (111, 112). Additional TFs, including STAT1, STAT2, and PPAR*γ*, have been implicated in the host response to secondary bacterial infection after influenza infection (113–115).

## Targeting TLR4 in Other Viral Respiratory Infections

The work of Imai et al. suggested that TLR4 signaling was important for induction of ALI by multiple viral and bacterial pathogens (19). While relatively few studies have been carried out to solidify this hypothesis, there are some that support it. Dengue virus (DENV) infection is endemic in many countries with approximately 400 million infections yearly (116). DENV causes a range of disease symptoms including classic dengue fever to severe hemorrhagic disease. Currently, there is no vaccine or therapy for DENV infection. The nonstructural protein 1 (NS1) of DENV can be either membrane-associated or found as a secreted, lipid-associated and soluble hexameric form (117). The soluble NS1 (sNS1) has been shown to directly activate TLR4 (as a PAMP) in both human peripheral blood mononuclear cells (PBMCs) and mouse bone marrow-derived macrophages. The induction of pro-inflammatory cytokines by DENV sNS1 was lost in TLR4^-/-^, but not TLR2^-/-^ macrophages, and could be blocked by inhibiting TLR4 signaling with a TLR4 antagonist, *Rhodobacter sphaeroides* LPS (Rs-LPS), in human PBMCs. Capillary vascular leakage induced by NS1 in an *in vivo* model of DENV was also inhibited with the TLR4 antagonist *Rhodobacter sphaeroides* LPS (Rs-LPS) or with a neutralizing anti-TLR4 antibody (118).

Ebola virus (EBOV) made a significant impact during its re-emergence between 2013-2016 (119, 120) and continues to be of epidemiological concern for central African countries. EBOV infection fatalities are associated with significant levels of pro-inflammatory cytokines and chemokines (121). Younan et al. reported that treatment of mice with Eritoran immediately following a lethal challenge with EBOV resulted in increased protection without improving weight loss or clinical scores (79) in contrast to Eritoran treatment of mice infected with influenza (24). However, Eritoran treatment of EBOV-infected mice did significantly decrease the levels of certain cytokines and chemokines (79). Human Immunodeficiency Virus (HIV), another globally important virus, is known to dysregulate the immune system with the loss of T cell proliferation, shifting Th1 cells to Th2 cells and associated cytokines, and high levels of secreted IL-10 (122–126). The HIV Tat protein has been shown to play a role in the host immune system modulation by acting at the cell surface to stimulate cytokine secretion, particularly IL-10, on monocytes and macrophages (127, 128). The N-terminal sequence of Tat was reported to interact with TLR4 (129). Eritoran inhibited HIV-1 Tat-dependent IL-10 induction in PBMCs and the physical and functional interaction was further confirmed by using a neutralizing anti-TLR4 antibody (130).

## Do Differences in Genetic Backgrounds of Mice Make a Difference?

Studies by Nhu et al. (20) and Shirey et al. (24) showed that mice with a targeted mutation in TLR4 (TLR4^-/-^ mice; kindly provided by Shizuo Akira (131) and extensively backcrossed to C57BL/6J mice) were resistant to PR8 and other strains of human influenza. More recently, using a CRISPR/Cas9 approach, mice were engineered onto a C57BL/6J background that express two TLR4 SNPs (D298G and N397I) that are homologous to the common TLR4 SNPs found in humans (D299G and T399I) (132) and have been reported to result in LPS-hyporesponsiveness (133). As expected, the “TLR4-SNP” mice were shown to be LPS-hyporesponsive *in vivo* and *in vitro*, but were more responsive to LPS than Akira’s TLR4^-/-^ strain backcrossed >12 times to C57BL/6J mice. When challenged with influenza PR8 (LD_90_), wildtype mice succumbed to infection, while TLR4^-/-^ mice were refractory (p<0.0001) (132), as previously reported (24, 25). The PR8-infected TLR4-SNP mice, however, showed significantly reduced lethality, but were somewhat more susceptible than TLR4^-/-^ mice. Increased resistance of the TLR4-SNP mice to PR8 infection was confirmed with respect to decreased lung pathology, as well as diminished cytokine gene and protein expression (132).

In contrast, in two other reports (134, 135), a different *Tlr4* mutant mouse strain, B6.B10ScN-Tlr4lps-del/JthJ, was shown to succumb to infection with the mouse-adapted A/WSN/33 strain and also to the PR8 strain of influenza, leading to the conclusion that loss of TLR4 did not protect against influenza-induced lethality (135). It is important to emphasize that the *Tlr4* mutation in the B6.B10ScN-Tlr4lps-del/JthJ strain is not the result of a targeted mutation; rather, this mouse strain was derived from a spontaneous deletion within *Tlr4* that occurred in the C57BL/10ScN strain between 1947 and 1961 (136). The B6.B10ScN-Tlr4lps-del/JthJ strain originated from the C57BL/10ScN colony at NCI Frederick (NIH), was transferred to the University of Texas Southwest Medical Center where it was backcrossed for 5 generations onto a C57BL/6 background. The strain was returned to Jackson Labs in 2008 where it was backcrossed to C57BL/6J for one additional generation during re-derivation (137). Although B6.B10ScN-Tlr4lps-del/JthJ strain used for these studies was indeed LPS-unresponsive, it is entirely possible that it carries background genes derived from the C57BL/10ScN parental strain that differ from C57BL/6J inbred strain and contribute to influenza sensitivity, since minor mutations in closely related strains are known to result in significant changes in phenotypes. For example, the progenitor strain, C57BL/10ScN, and its successor, C57BL/10ScCR, both express the deletion in *Tlr4*; however, the latter strain possesses an additional point mutation in the gene encoding the IL-12Rβ2, causing it to express a distinct phenotype affecting IFN-*γ* production in response to infection (138). This has also been observed among closely related C57BL/6 substrains (139). To further evaluate the role of distinct strain backgrounds and the role of TLR4 in influenza-induced lethality, wild-type C3H/HeOuJ and C3H/HeJ mice, the latter strain shown to express a point mutation in the TIR domain of TLR4 that resulted in a loss of TLR4-mediated signaling (140, 141), were challenged with mouse-adapted influenza H1N1 PR8. As previously reported in C57BL/6J *versus* TLR4^-/-^ mice (20, 24), the TLR4 signaling-deficient C3H/HeJ strain was significantly more resistant to PR8-induced lethality than the closely related, LPS-responsive C3H/OuJ strain from which it was derived when challenged with either a sublethal (p = 0.0118) or lethal dose (p = 0.0004) of influenza strain PR8. Thus, the increased resistance of TLR4^-/-^ and C3H/HeJ mice, coupled with the ability of many distinct TLR4 antagonists to block influenza-mediated cytokine production, ALI, and lethality, strongly support a critical role for TLR4 signaling in influenza-induced disease.

## Concluding Remarks

During the past year, we have all become too familiar with COVID-19 pandemic that has led to ALI, as well as the more severe acute respiratory distress syndrome (ARDS) and the deaths of millions worldwide. In support of the observation that TLR4^-/-^ mice are extremely refractory to influenza infection (20, 24), other studies have subsequently shown that many TLR4 antagonists, that act by a variety of distinct mechanisms to prevent signaling all block influenza-induced lethality. This is independent of the influenza virus strain used and has been confirmed in other rodent models of influenza infection. More importantly, the interference with TLR4 signaling by agents like Eritoran has shown promise with other viruses, again supporting the importance of TLR4 in other virus-induced diseases. The striking similarities between influenza- and SARS-CoV-2 induced ARDS caused by a cytokine storm leading to loss of homeostasis and multiorgan failure, has been referred to as “viral sepsis” (142). By understanding the mechanisms by which the virus-induced “cytokine storm” is mitigated, we expect to discover therapeutic targets in the influenza model that will be applicable also to patients with severe SARS-CoV-2 and other virus-induced lung diseases. A recent study reported that the SARS-CoV-2 trimeric spike (S) protein directly interacted with and activated TLR4 *in vitro* in human and murine macrophage-like cell lines, and in bone marrow-derived macrophages from wildtype, but not TLR4^-/-^, mice (143). Moreover, treatment of cells with Resatorvid (TAK-242), that blocks MyD88 interaction with TLR4 (31), also blocked S protein-induced *Il1b* mRNA (143). However, the finding that inhibition of either MD2 or CD14 blunted gene expression suggests that the S protein may be binding not only to TLR4, as suggested by their binding data, but also to these TLR4 co-receptors. Future studies to confirm this interesting report will be warranted to define more clearly the possible interaction between S protein and the TLR4 signaling complex. Collectively, insights into key molecular interactions that underlie host-pathogen interactions support the novel concept that host-directed therapeutics that selectively target interactions between PRRs and PAMPs/DAMPs, or the signaling pathways they initiate, might serve as potentially uniquely effective therapeutic strategies that could be mobilized to target multiple infectious pathogens.

## Author Contributions

KS, JB, and SV contributed to the original writing and editing of the manuscript. All authors contributed to the article and approved the submitted version.

## Funding

This work was supported by AI123371 (SV), AI125215 (SV/JB), and AI159507 (KS).

## Conflict of Interest

JB was employed by Sigmovir Biosystems, Inc.

The remaining authors declare that the research was conducted in the absence of any commercial or financial relationships that could be construed as a potential conflict of interest.

## Abbreviations

ALI, Acute lung injury; ARDS, Acute respiratory distress syndrome; DAMP, Danger-associated molecular pattern; DENV, Dengue virus; EGCG, Epigallocatechin-3-gallate; EBOV, Ebola virus; GABA, *γ*-amibobutric acid; GRP, Gastrin releasing peptide; HMGB1, High-mobility group box 1; HIV, Human Immunodeficiency virus; IAV, Influenza A virus; LRR, Leucine-rich repeats; LPS, Lipopolysaccharide; LSW, Liu Shen Wan; MDA, Malondialdehyde; MAC, Monoacetylcurcumin; MRSA, Multi-drug resistant *Staphylococcus aureus*; NAC, N-acetyl-L-cystine; NA, Neuraminidase; NEB, Neuroendocrine bodies; OxPAPC, Oxidized 1-palmitoyl-2-arachidonoyl-phosphatidycholine; PAMP, Pathogen-associated molecular pattern; PRR, Pattern recognition receptors; PBMC, Peripheral blood mononuclear cells; ROS, Reactive oxygen species; Rs-LPS, *Rhodobacter sphaeroides* LPS; SNP, Single nucleotide polymorphisms; (S) protein, Spike protein; SFN, Sulforaphane; SP, Surfactant protein; TIR, Toll-IL-1 receptor resistance; TLR, Toll-like receptor; TF, Transcription factor.
